# A System of Surgery: Pathological, Diagnostic, Therapeutic and Operative

**Published:** 1862-02

**Authors:** 


### Book Notices.

A System of Surgery: Pathological, Diagnostic, Therapeutic, and Operative. By Samuel D. Gross, M. D., Professor of Surgery in the Jefferson Medical College of Philadelphia, etc., etc. Illustrated by twelve hundred and twenty-seven engravings. Second edition, much enlarged and carefully revised. In two volumes. Blanchard & Lea: Philadelphia. 1862.

An unanimous verdict of praise from the medical profession of every civilized country, is not often accorded to any living author. Not until the shining wand of the angel Azrael has bound with its silent spell, the busy brain, do we usually give to our Hunters, our Sydenhams and our Rushes, the just meed of their labors. At best, while alive, a foreign popularity, the alien tribute of a "contemporaneous posterity," is the least rarely granted,—as though distance, if but of space, were absolutely necessary to the proper appreciation of intellectual greatness. The Pyramids may not be judged by him who stands in their shadow.

But to the eminent surgeon, whose volumes lie before us. Fame has not been tardy. His own countrymen have not left it to other nations to discover his claims to pre-eminence; and other nations, with a generous speed, have hastened to confer upon him the merit of the soundest, the *first* surgical author ity of his time. And the glory is reflected from the professor to the profession,—so that, despite the carping sneer against the New World and its people, which Sydney Smith moulded into an historic query,—American surgery is, to-day, head and shoulders above its brethren of France and Great Britain, as well in vastness of resources as in careful and scrupulous conservation of limb and life. And Prof. Gross is the chosen and approved exponent of the American system,—rapidly becoming, through this, his *magnum opus*, the cosmopolitan.

That a new edition should so soon be called for, is not, to us, so surprising, as that so much, valuable and interesting, could be found to add to its pages. Every chapter has been revised, introducing a large amount of new matter, all of an essentially practical character; and the illustrations have been increased from nine hundred and odd, to over twelve hundred. The subject of *Gunshot Wounds*, so fearfully important, at this time, has received especial attention, and this section is now one of the fullest and most practical in the book. In fact, we know of no one work that can at all compete with this, in value and importance to the military surgeon, as well as to the general practitioner and surgeon in civil life.

The familiar and popular text-book of Robert Druitt derives one of its chief advantages, according to an eminent reviewer, from "the fact that it was first written when the author was still a student, before he had his own particular experience to relate, and was willing and desirous of writing down simply the principles and practice of general experience." The work under consideration derives its chief value from a directly opposite fact, *i. e.*, that it is the fruit of a third of a century's study and experience, the ripe harvest of a rare combination of excellent judgment, extensive and enlightened research, and varied practical observation. That the intentions of the author, as shadowed forth in the following extract from the preface to the first edition, have been fully carried out, its popularity and wide-spread reputation fairly prove:—

The object of this work is to furnish a systematic and comprehensive treatise on the science and practice of surgery, considered in the broadest sense; one that shall serve the practitioner as a faithful and available guide in his daily routine of duty. It has been too much the custom of modern writers on this department of the healing art, to omit certain topics altogether, and to speak of others at undue length, evidently assuming that their readers could readily supply the deficiencies from other sources, or that what has been thus slighted is of no particular practical value. My aim has been to embrace the whole domain of surgery, and to allot to every subject its legitimate claim to notice in the great family of external diseases and accidents. How far this object has been accomplished, is not for me to determine. It may safely be affirmed, however, that there is no topic, properly appertaining to surgery, that will not be found to be discussed, to a greater or less extent, in these volumes. If a larger space than is customary has been devoted to the consideration of inflammation and its results, or the great principles of surgery, it is because of the conviction, grounded upon long and close observation, that there are no subjects so little understood by the general practitioner. Special attention has also been bestowed upon the discrimination of diseases; and an elaborate chapter has also been introduced on general diagnosis.

The publishers have faithfully done their share in the production of an American volume of which we are all proud. The type, though smaller than in the first edition, is beautifully clear and distinct, the illustrations are excellent, both in the engraving and printing, and the binding is strong and substantial. By the use of the smaller type, the increase of matter is admitted, with no additional bulk,—indeed, the present volumes are less cumbrous than the old ones, and number some two hundred pages less.

